# Who Suffers From Pharmaceutical Poverty and What Are Their Needs? Evidence From a Spanish Region

**DOI:** 10.3389/fphar.2021.617687

**Published:** 2021-04-20

**Authors:** Maria Rubio-Valera, Silvia Marqués-Ercilla, M Teresa Peñarrubia-María, Rosa M. Urbanos-Garrido, Carme Borrell, Jordi Bosch, Alba Sánchez-Viñas, Ignacio Aznar-Lou

**Affiliations:** ^1^ Research and Teaching Unit, Parc Sanitari Sant Joan de Déu, Sant Boi de Llobregat, Spain; ^2^ The Biomedical Research Centre Network for Epidemiology and Public Health (CIBERESP), Madrid, Spain; ^3^ Centre d’Atenció Primària Bartomeu Fabrés Anglada, Direcció d'Atenció Primària Costa de Ponent, Institut Català de la Salut, Gavà, Spain; ^4^ Unitat de Suport a la Recerca Costa de Ponent, Fundació Institut Universitari per a la recerca a l'Atenció Primària de Salut Jordi Gol i Gurina (IDIAPJGol), Cornellà de Llobregat, Spain; ^5^ Department of Applied Economics, Public Economics and Political Economy, The Complutense University of Madrid, Madrid, Spain; ^6^ Agència de Salut Pública de Barcelona, Barcelona, Spain; ^7^ Pharmaceutical Bank, Barcelona, Spain

**Keywords:** healthcare disparities (MeSH), primary care (MeSH), pharmaceutical poverty, pharmaceutical preparations, health services accessibility

## Abstract

**Background:** Pharmaceutical poverty occurs when a patient cannot afford the cost of prescribed medication and/or medical products. Nonprofit organizations are covering the cost of medication to those patients in some contexts. The aim of the study was to describe the population of beneficiaries of the PB, a nongovernmental organization based on the primary healthcare system, which provides free-of-charge access to medicines and their utilization pattern of medicines and healthcare products.

**Methods:** This was an observational study using PB beneficiary data collected between November 2017 and December 2018 in Catalonia. The Catalan Health Service provided information from the general population. A descriptive analysis of the beneficiaries’ characteristics was conducted and compared to the general population.

**Results:** The beneficiaries (N = 1,206) were mainly adults with a low level of education, unemployed, with functional disability, and with ≥1 child. Compared with the general population, the beneficiaries were older, had a lower level of education, showed a higher prevalence of functional disability, were less likely to be Spanish, and were more likely to be divorced and unemployed. The beneficiaries were polymedicated, and most were using medication related to the nervous (79%), musculoskeletal (68%), and cardiovascular system (56%) and alimentary tract and metabolism (68%). Almost 19% of beneficiaries used healthcare products. Female beneficiaries were older and more likely to be divorced or widowed, employed, and with children. Compared to men, women were more likely to use medicines for pain and mental disorders. The pediatric group used medications for severe, chronic conditions (heart diseases, autoimmune diseases, conduct disorders, and attention deficit hyperactivity disorder).

**Conclusion:** Patients with severe, chronic, and disabling conditions are affected by pharmaceutical poverty. While the system of copayment remains unchanged, family physicians and pediatricians should explore economic barriers to treatment and direct their patients to resources that help to cover the cost of treatment.

## Introduction

Universal healthcare coverage aims to ensure that everyone can use necessary health services without experiencing financial hardship. However, medicines are subject to out-of-pocket payments worldwide (Thomson et al., 2019). The cost of medicines has been systematically identified as a factor that limits adherence to medications, mainly for disadvantaged populations (Choudhry et al., 2014; Simoens and Sinnaeve, 2014; Terraneo et al., 2014; Banerjee et al., 2016; Sinnott et al., 2016; Aznar-Lou et al., 2018). The failure to effectively use prescription medicines can lead to worse health status and higher costs to the healthcare system (Kesselheim et al., 2015). Nonadherence worsens health outcomes and increases the risk of mortality and hospitalization (Xu et al., 2017; Kim et al., 2018). Greater use of medical services and productivity losses are also consequences of medication nonadherence, which places a significant cost burden on healthcare systems (Aznar-Lou et al., 2017; Cutler et al., 2018). Nonadherence is higher when more expensive medicines are prescribed and when copayment is applied (Kazerooni et al., 2013; González López-Valcárcel et al., 2017; Aznar-Lou et al., 2018); interventions that reduce the copayment contributions or exempt the patient from payment have effectively proven to reduce nonadherence (Chernew et al., 2008; Maciejewski et al., 2010).

The World Health Organization (WHO) defines catastrophic health spending as those health expenditures “greater than or equal to 40% of a household’s non-subsistence income, i.e., income available after basic needs have been met” (Xu et al., 2005). In low- and middle-income countries, the cost of medicines can be up to 60% of total healthcare expenditure (Cameron et al., 2009) and family members becoming ill can have catastrophic consequences (Barasa et al., 2017; Datta et al., 2019). According to a recent report by the WHO, medicines are the main cause of out-of-pocket payments incurred by households with catastrophic health spending (i.e., households that cannot afford to meet basic needs like food, housing, and heating because of a health-related condition) also in high-income countries (Thomson et al., 2019). Catastrophic health spending may lead to or deepen poverty, undermine health, and exacerbate health and socioeconomic inequalities (Thomson et al., 2019).

In Spain, the National Health System (NHS) is taxpayer funded, covers residents and foreign nationals, and, with a few exceptions, is free at the point of delivery. In Catalonia, one of the most populated regions in Spain, where 7.6 million people live, there are 371 publicly financed primary care centers that manage most of the prescriptions (Bolíbar et al., 2012). Prescription and over-the-counter pharmacological treatments are dispensed in privately managed community pharmacies. Patients pay a part of the cost of prescription medication, with the share dependent on the medication and patients’ characteristics. The copayment applies to pensioners and nonpensioners and ranges from 0 to 60% depending on the annual income, with a 10% reduced contribution for some medications (most chronic treatments) (España’s, 2012). Upper limits to copayments are set only for pensioners, ranging from around 8 to 62 euros per month, and all patients pay 100% of the cost of medicines not covered by the NHS. Over 70% of primary care attendees have an assigned copayment of 40% or 10% (Aznar-Lou et al., 2018). Although some particular groups are exempt from copayment (disabled patients, people receiving noncontributory pensions, unemployed people whose benefits expired, or beneficiaries of social integration subsidies), in 2018, 3% of Spaniards reported not having taken a medication prescribed in a public healthcare center because they could not afford it (de Investigaciones Sociológicas, 2018).

In response to this situation, in recent years, several nongovernmental organizations (NGOs) have provided free-of-charge access to medicines. This is the case of the NGO Pharmaceutical Bank (PB) which, in 2015, launched a pilot program in Barcelona—the Social Medicine Fund (SMF)—to fight against what was called “pharmaceutical poverty.” The NGO, in collaboration with the Catalan Health Institute (which manages 80% of Catalan primary care centers) and the local council, began to cover the cost of prescribed medication and medical products to those patients attended in primary care centers who could not afford their treatments. Since then, the program has been further extended and is now available not only in the city of Barcelona, but also in eight other cities in the metropolitan area and some cities outside the region of Catalonia.

In order to benefit from the SMF, patients must first visit primary care social workers who corroborate their medical needs and verify their financial situation, through inspection of tax returns, payrolls and rent or mortgage receipts, etc. Social workers provide the beneficiaries with a list of partner community pharmacies where they can get the medicines free of charge. The beneficiaries have to fulfill the following criteria: 1) to be attended in a Catalan Health Institute primary care center, 2) to require a treatment for at least 6 months which has a monthly cost of 20 Euros or more, and 3) to be in a situation of financial hardship (defined as a monthly household income, after discounting rent or mortgage, lower than €550 for one person, €670 for two people, or €670 plus €75 per additional person).

The SMF covers the patients’ total treatment cost for a period of six months, which may be renewed if the conditions have not changed. The aid covers the cost of prescription medicines and healthcare products. In exceptional cases, when it is clinically relevant, the SMF also covers the cost of over-the-counter medicines. The NGO Pharmaceutical Bank is partnered with Catalan Health Institute primary care professionals and pharmacists from several community pharmacies. In Catalonia in 2018, 124 primary care centers and 407 community pharmacies were collaborating with the SMF.

There is some evidence on inadequate access to medicines in developing countries (Leisinger et al., 2012; Chan, 2017), but, to the best of our knowledge, no previous study has provided information on the use of medicines by patients in a situation of pharmaceutical poverty or on the characteristics of these patients in western societies, although new tools to measure access to medicines, which would allow for comparisons within countries, are being developed (Garcia et al., 2019). This article gives a detailed description of SMF beneficiaries and of their pattern of utilization of medicines and healthcare products, which is compared with the pattern of use in the general population in Catalonia.

## Methods

### Design

This was an observational study using data from the Pharmaceutical Bank NGO database, collected between November 2017 and December 2018 in Catalonia.

### Variables

Our database includes information on beneficiaries’ characteristics, including gender (female and male), age, educational level (without formal education and primary, secondary, or higher education), nationality (Spanish and other), presence of functional disability, and assigned percentage of copayment (10, 40, 50, or 60%). In the adult population (≥18 years), civil status (married, single, divorced, widowed, or other), employment status (active, unemployed with benefits, pensioner or beneficiary of social assistance, or unemployed without benefits), and number of children were also registered.

Individuals were divided into three age groups (0–14, 15–64, and ≥65 years). In Spain, patients are considered pediatric from 0 to 14 years (and treated in specific services) and people retire at the age of 65, when they began to present more chronic conditions. This classification was made to allow for comparison with data from the Catalan Health Department. The number of children was also categorized (0, 1, 2, 3, 4, or more).

Permanence in the SMF was estimated by taking into account the number of renewals and classified as no renewal (6 months), one renewal (6–12 months), or more.

Medicines provided by the NGO were classified using the Anatomical Therapeutic Chemical (ATC) system. The selected healthcare products were those most frequently used: wound dressings, surgical wound dressings, varicose vein stockings and socks, inhalation chambers, diapers, and allergen-specific immunotherapy, with a final category left for other products.

The Catalan Health Survey 2018 (ESCA; Enquesta de Salut de Catalunya) was used as a source of data from the general Catalan population. ESCA is an official survey consisting of a multistage probability sample representative of noninstitutionalized residents of Catalonia, which includes information about personal characteristics, lifestyles, use of health services, and health indicators. Finally, the Catalan Health Service provided information on the use of publicly financed medicines in 2018 for the total Catalan population.

### Analysis Strategy

A descriptive analysis of the beneficiaries’ characteristics was conducted using means for continuous variables and proportions for categorical variables. Using data from the ESCA, the characteristics of the general Catalan population were also described. Probability weights were applied to ensure representativeness.

First, we calculated the proportion of beneficiaries that received at least one medication by anatomical group and therapeutic subgroup, and the proportion of beneficiaries that received at least one healthcare product. Then, the overall use of medicines and healthcare products covered by the NGO and CatSalut was described (frequency and proportion of the total number of medicines—anatomical groups and therapeutic subgroups—and healthcare products were estimated). The profiles of medicine and healthcare product use are shown by gender and age group (0–14, 15–64, and ≥65 years).

## Results

### Characteristics of Beneficiaries

Between November 2017 and December 2018, 1,206 people benefited from the SMF and 227 (18.8%) and 147 (12.2%) renewed the help once and twice, respectively. Almost 96% (1,154) of beneficiaries had an assigned copayment rate of 40% (corresponding to nonpensioners with annual incomes up to 18,000€), 3.1% (Evans-Lacko et al., 2013) had a copayment rate of 10%, and the rest had a copayment rate of 50% or 60%.

Table 1 compares the sociodemographic characteristics of beneficiaries of the NGO Pharmaceutical Bank in Catalonia and those of the general Catalan population. Mean age was almost 49 years, and 52% of the beneficiaries were female. Most of the population was Spanish (62.1%), and a third presented functional disability. Among the adult population, most had primary or no formal education (68.3%), were unemployed without benefits (57.0%), and had up to two children. Most common civil status in the adult population was married (38.4%), followed by single (28.9%) and divorced or separated (23.1%).

**TABLE 1:** Sociodemographic characteristics of the population.

	General population^a^ (n = 4,827) % [SE]^b^	PB beneficiaries (n = 1,206) % (n)^b^	Female beneficiaries (n = 623)	Male beneficiaries (n = 583)
Mean age (SE)	42.3 (0.31)	48.9 (0.53)	51.0 (0.70)	46.6 (0.78)
Age group				
0–14 years old	15.6 (0.47)	8.6 (104)	6.7 (42)	10.8 (63)
15–64 years old	65.8 (0.67)	76.1 (918)	75.1 (468)	77.5 (452)
≥65 years old	18.7 (0.55)	15.3 (184)	18.1 (113)	11.7 (68)
Female	50.6 (0.75)	51.7 (623)	-	-
Functional disability	11.8 (0.46)	34.7 (419)	36.3 (226)	33.1 (193)
Nationality				
Spanish	85.7 (0.54)	62.1 (749)	64.0 (399)	60.0 (350)
Other	11.8 (0.50)	37.5 (452)	35.5 (221)	39.6 (231)
Spanish and other	2.5 (0.24)	Na	Na	Na
Missing		0.4 (5)	0.5 (3)	0.3 (2)
Adult population (≥18 years old)	(n = 3,600)	(n = 1,065)	(n = 570)	(n = 495)
Educational level				
Without education or primary education	19.6 (0.64)	68.3 (727)	70.5 (439)	70.2 (409)
Secondary education	58.5 (0.84)	25.5 (272)	24.1 (150)	24.2 (141)
Higher education	21.6 (0.72)	6.0 (64)	5.3 (33)	5.5 (32)
Missing	0.3 (0.09)	0.2 (2)	0.2 (1)	0.2 (1)
Civil status^c^				
Married	57.2 (1.25)	38.4 (409)	33.2 (189)	44.4 (220)
Single	28.8 (1.14)	28.9 (308)	25.4 (145)	32.9 (163)
Divorced or separated	6.0 (0.49)	23.1 (246)	27.2 (155)	18.4 (91)
Widowed	8.1 (0.69)	6.6 (70)	10.2 (58)	2.4 (12)
Other	-	2.8 (30)	3.9 (22)	1.6 (8)
Missing	-	0.2 (2)	0.2 (1)	0.2 (1)
Employment status				
Active	55.7 (0.84)	19.0 (138)	14.9 (85)	10.7 (53)
Unemployed with benefits	3.4 (0.32)	15.4 (164)	13.2 (75)	18.0 (89)
Pensioner or beneficiary of social help	6.2 (0.38)	14.5 (154)	15.8 (90)	12.9 (64)
Unemployed without benefits	2.7 (0.28)	57.0 (607)	56.0 (319)	58.2 (288)
Housewife/husband	11.0 (0.42)	Na	Na	Na
Student	3.8 (0.32)	Na	Na	Na
Other	Na	Na	Na	Na
Missing	0.3 (0.09)	0.0 (2)	0.2 (1)	0.2 (1)
Number of children				
0	Na	29.1 (310)	22.8 (130)	36.4 (180)
1	Na	18.2 (194)	20.2 (115)	16.0 (79)
2	Na	26.1 (278)	26.8 (153)	25.3 (125)
3	Na	11.9 (127)	14.6 (83)	8.9 (44)
4 or more children	Na	14.7 (156)	15.6 (89)	13.5 (67)

^a^ Data from the Catalan Health Survey (Enquesta de Salut de Catalunya (ESCA)) in 2018.

^b^ With the exception of mean age (mean (SE)).

^c^ Data from the Catalan Health Survey (ESCA) in 2014 (this data was collected the previous year ) (n = 1804).

PB: Pharmaceutical Bank; Na: data not available or not applicable.

Bold numbers indicate statistically significant differences (p < 0.05) between male and female beneficiaries on Students’ *t* test for mean age and chi-squared tests for categorical variables.

Compared with the general Catalan population, the population of beneficiaries was older, had a lower level of education, showed a higher prevalence of functional disability, was less likely to be Spanish, and was more likely to be divorced and unemployed.

In comparison with male beneficiaries, female beneficiaries were older, more likely to be active and divorced or widowed, and more likely to have children.

### Patterns of Drugs Use

#### Proportion of Beneficiaries With At Least One Medicine From Each Group

Tables 2 and 3 show the proportion of beneficiaries using at least one healthcare product or medicine from each anatomical group and therapeutic subgroup (therapeutic subgroups and type of healthcare product are only shown if they have been used by at least 20% of beneficiaries; full data are shown in Supplementary Tables 1 and 2). This information was not available for the Catalan general population.

**TABLE 2:** Proportion of beneficiaries that received at least one medicine or healthcare product overall and for gender, % (N).

Medicines, anatomical group (ATC level 1), and therapeutic group (ATC level 2)^a^	Total (n = 1,206)	Females (n = 623)	Males (n = 583)
Alimentary tract and metabolism (A)	67.7 (817)	73.8 (460)	61.2 (357)
Drugs for acid-related disorders (A02)	51.1 (616)	55.9 (348)	46.0 (268)
Drugs used in diabetes (A10)	26.0 (314)	24.7 (154)	27.4 (160)
Blood and blood-forming organs (B)	41.0 (495)	38.2 (238)	44.1 (257)
Antithrombotic agents (B01)	30.8 (371)	24.4 (152)	37.6 (219)
Cardiovascular system (C)	55.9 (674)	53.9 (336)	58.0 (338)
Beta blocking agents (C07)	20.0 (241)	13.2 (82)	27.3 (159)
Agents acting on the renin-angiotensin system (C09)	36.0 (434)	35.0 (218)	37.1 (216)
Lipid-modifying agents (C10)	38.6 (466)	32.9 (205)	44.8 (261)
Dermatologicals (D)	20.4 (246)	25.0 (156)	15.4 (90)
Genitourinary system and sex hormones (G)	11.4 (137)	11.2 (70)	11.5 (67)
Systemic hormonal preparations, excluding sex hormones and insulin (H)	17.3 (209)	23.4 (146)	10.8 (63)
Anti-infectives for systemic use (J)	25.3 (305)	28.1 (175)	22.3 (130)
Antibacterials for systemic use (J01)	23.6 (284)	26.0 (162)	20.9 (122)
Antineoplastic and immunomodulating agents (L)	7.1 (86)	10.1 (63)	4.0 (23)
Musculoskeletal system (M)	68.1 (821)	38.8 (242)	24.5 (143)
Anti-inflammatory and antirheumatic products (M01)	26.4 (318)	34.0 (212)	18.2 (106)
Nervous system (N)	78.9 (952)	84.3 (525)	73.2 (427)
Analgesics (N02)^b^	56.0 (675)	65.8 (410)	45.5 (265)
Other opioids (N02AX)	17.1 (206)	22.0 (137)	11.8 (69)
Pyrazolones (N02BB)	18.9 (228)	23.9 (149)	13.6 (79)
Anilides (N02BE)	44.4 (535)	51.4 (320)	36.9 (215)
Antiepileptics (N03)^b^	26.5 (319)	29.1 (181)	23.7 (138)
Other antiepileptics (N03AX)	22.2 (268)	26.0 (162)	18.2 (106)
Psycholeptics (N05)	43.5 (525)	46.7 (291)	40.1 (234)
Psychoanaleptics (N06)	41.9 (505)	49.1 (306)	34.1 (199)
Antiparasitic products, insecticides, and repellents (P)	1.6 (19)	1.8 (11)	1.4 (8)
Respiratory system (R)	32.1 (387)	34.0 (212)	30.0 (175)
Drugs for obstructive airway diseases (R03)	21.3 (257)	21.4 (133)	21.3 (124)
Sensory organs (S)	13.4 (161)	15.9 (99)	10.6 (62)
Various (V)	1.8 (22)	2.1 (13)	1.5 (9)
Healthcare products	18.7 (226)	20.7 (129)	16.6 (97)

^a^ Only the therapeutic groups and type of healthcare products that have been used by at least 20% of the beneficiaries are shown. Supplementary files show full data.

^b^ For analgesics and antiepileptics, which are highly heterogeneous, the anatomical therapeutic chemical level is shown if <10% of the beneficiaries have used medications.

**TABLE 3:** Proportion of beneficiaries that received at least one medicine or healthcare product for age groups, % (N).

Medicines, anatomical group (ATC level 1), and therapeutic group (ATC level 2)^a^	≤14 (n = 104)	15–64 (n = 918)	≥65 (n = 184)
Alimentary tract and metabolism (A)	13.3 (14)	70.6 (647)	84.8 (156)
Drugs for acid-related disorders (A02)	5.7 (6)	53.5 (491)	64.7 (119)
Drugs used in diabetes (A10)	1.0 (1)	25.6 (235)	42.4 (78)
Blood and blood-forming organs (B)	7.6 (8)	41.3 (379)	58.7 (108)
Antithrombotic agents (B01)	0 (0)	30.2 (277)	51.2 (94)
Cardiovascular system (C)	9.5 (10)	55.6 (510)	83.7 (154)
Diuretics (C03)	0 (0)	14.5 (133)	25.5 (47)
Beta blocking agents (C07)	2.9 (3)	20.6 (189)	26.6 (49)
Calcium channel blockers (C08)	1.0 (1)	13.4 (123)	25.0 (46)
Agents acting on the renin-angiotensin system (C09)	1.9 (2)	34.6 (317)	62.5 (115)
Lipid-modifying agents (C10)	0 (0)	38.6 (354)	60.9 (112)
Dermatologicals (D)	12.4 (13)	20.5 (188)	24.5 (45)
Genitourinary system and sex hormones (G)	1.9 (2)	10.9 (100)	19.0 (35)
Systemic hormonal preparations, excluding sex hormones and insulin (H)	10.5 (11)	17.9 (164)	18.5 (34)
Anti-infectives for systemic use (J)	19.1 (20)	26.7 (245)	21.7 (40)
Antibacterials for systemic use (J01)	18.1 (19)	24.9 (229)	19.9 (36)
Antineoplastic and immunomodulating agents (L)	5.7 (6)	7.2 (66)	7.6 (14)
Musculoskeletal system (M)	21.9 (23)	34.6 (317)	24.5 (45)
Anti-inflammatory and antirheumatic products (M01)	21.0 (22)	28.6 (262)	18.5 (34)
Nervous system (N)	61.0 (64)	80.3 (736)	82.6 (152)
Analgesics (N02)^b^	22.9 (24)	58.2 (534)	63.6 (117)
Other opioids (N02AX)	0	19.1 (175)	16.6 (31)
Pyrazolones (N02BB)	0	20.2 (185)	23.0 (43)
Anilides (N02BE)	21.2 (22)	44.7 (409)	55.6 (104)
Antiepileptics (N03)^b^	9.5 (10)	30.0 (275)	18.5 (34)
Other antiepileptics (N03AX)	3.9 (4)	25.4 (232)	17.1 (32)
Psycholeptics (N05)	28.6 (30)	46.1 (423)	39.1 (72)
Psychoanaleptics (N06)	33.3 (35 )	43.4 (398)	39.1 (72)
Antiparasitic products, insecticides, and repellents (P)	2.9 (3)	1.7 (16)	0 (0)
Respiratory system (R)	36.2 (38)	31.5 (289)	32.6 (60)
Drugs for obstructive airway diseases (R03)	26.7 (28)	20.0 (183)	25.0 (46)
Antihistamines for systemic use (R06)	20.0 (21)	13.6 (125)	9.8 (18)
Sensory organs (S)	12.4 (13)	11.2 (103)	24.5 (45)
Ophthalmologicals (S01)	11.4 (12)	9.7 (89)	23.8 (43)
Various (V)	0 (0)	2.2 (20)	1.1 (2)
Healthcare products	38.1 (40)	14.4 (132)	29.4 (54)
Diapers	10.5 (11)	4.8 (44)	21.2 (39)
Allergen-specific immunotherapy	21.0 (22)	1.4 (13)	0 (0)

^a^ Only the therapeutic groups and type of healthcare products that have been used by at least 20% of the beneficiaries are shown. Supplementary files show full data.

^b^ For analgesics and antiepileptics, which are highly heterogeneous, the anatomical therapeutic chemical level is shown if <10% of the beneficiaries have used medications.

The medications most commonly consumed by the beneficiaries were those related to the “nervous system” (78.9%), followed by those related to the “musculoskeletal system” (68.1%), “alimentary tract and metabolism” (67.7%), and “cardiovascular system” (55.9%). Disaggregation by therapeutic subgroup indicated that a large proportion of the population received medication for pain-related disorders [analgesics (56.0%); anti-inflammatory and antirheumatic products (26.4%); and other antiepileptics (22.2%)], gastrointestinal disorders [drugs for acid-related disorders (51.1%) and drugs for functional gastrointestinal disorders (7.6%)], mental health–related disorders [psycholeptics (43.5%) and psychoanaleptics (41.9%)], cardiovascular diseases [lipid-modifying agents (38.6%), agents acting on the renin-angiotensin system (36.0%), antithrombotic agents (30.8%), beta blocking agents (20.0%), diuretics (14.9%), and calcium channel blockers (14.1%)], diabetes [drugs used in diabetes (26.0%)], and allergies and asthma [drugs for obstructive airway diseases (21.3%) and antihistamines for systemic use (13.6%)]. Almost 19% of NGO beneficiaries used at least one healthcare product.

Males and females followed a similar pattern (Table 2) although, compared with male beneficiaries, females were more likely to use medication for pain-related disorders (e.g., 65.8 vs. 45.5% in analgesics), gastrointestinal disorders (e.g., 55.9 vs. 46.0% in drugs for acid-related disorders), and mental health disorders (e.g., 49.1 vs. 34.1% in psychoanaleptics) and less likely to use medications for cardiovascular diseases (e.g., 32.9 vs. 44.8% in lipid-modifying agents) and diabetes (e.g., 24.7 vs. 27.4% in drugs used in diabetes).

Among children (Table 3), the most frequently used medicines were those for the “nervous system” (61.0%), “respiratory system” (36.2%), and “musculoskeletal system” (21.9%). There was a highly prevalent use of medicines for the “nervous system” (80.3%), “alimentary tract and metabolism” (70.6%), and “cardiovascular system” (55.6%) among beneficiaries aged between 15 and 64 years. Over 80% of the oldest group of beneficiaries was using medications for the “alimentary tract and metabolism,” “cardiovascular system,” and “nervous system.”

### Volume of Medicines and Healthcare Products Covered by the PB and the CatSalut

Tables 4 and 5 (and Supplementary Tables 3 and 4) show the relative weight of each medicine and healthcare product with respect to the total number of units of medicines and healthcare products, for PB beneficiaries and for the whole Catalan population.

**TABLE 4:** Proportion of medicines and products used by the beneficiaries and the general population, % (N).

	Total		Males		Females	
	PB	Catalonia	PB	Catalonia	PB	Catalonia
Medicines, anatomical group (ATC level 1) and therapeutic group (ATC level 2)^a^	48,747	137,378,189	21,225	59,400,000	27,522	77,500,000
Alimentary tract and metabolism (A)	16.0 (7,788)	14.3 (19,644,551)	15.9 (3,375)	14.5 (8,632,772)	16.0 (4,413)	14.1 (10,900,000)
Drugs for acid related disorders (A02)	5.7 (2,777)	6.2 (8,547,897)	5.4 (1,136)	6.0 (3,571,192)	6.0 (1,641)	6.4 (4,950,959)
Drugs used in diabetes (A10)	6.3 (3,081)	4.9 (6,777,457)	7.6 (1,621)	6.4 (3,814,420)	5.3 (1,460)	3.8 (2,945,215)
Blood and blood forming organs (B)	6.3 (3,052)	6.1 (8,310,079)	8.1 (1711)	7.0 (4,163,092)	4.9 (1,341)	5.3 (4,123,536)
Cardiovascular system (C)	19.1 (9,320)	25.0 (34,405,442)	24.9 (5,281)	29.0 (17,200,000)	14.7 (4,039)	22.1 (17,100,000)
Lipid modifying agents (C10)	5.8 (2,836)	6.6 (9,019,823)	7.7 (1,636)	8.0 (4,767,132)	4.4 (1,200)	5.5 (4,229,100)
Dermatologicals (D)	2.0 (951)	1.7 (2,368,761)	1.4 (304)	1.8 (1,081,384)	2.4 (647)	1.7 (1,274,461)
Genito urinary system and sex hormones (G)	1.4 (668)	2.7 (3,769,794)	1.7 (369)	4.3 (2,538,678)	1.1 (299)	1.6 (1,219,365)
Systemic hormonal preparations, excl. Sex hormones and insulins (H)	1.5 (720)	1.8 (2,510,611)	0.7 (146)	1.3 (763,523)	2.1 (574)	2.2 (1,736,597)
Antiinfectives for systemic use (J)	2.6 (1,247)	3.4 (4,598,444)	2.2 (473)	3.3 (1,942,406)	2.8 (774)	3.4 (2,610,900)
Antineoplastic and immunomodulating agents (L)	2.1 (1,029)	1.0 (1,364,320)	1.7 (369)	0.9 (556,179)	2.4 (660)	1.0 (804,895)
Musculo-skeletal system (M)	3.9 (1878)	1.5 (2,085,977)	3.0 (644)	4.4 (2,619,746)	4.5 (1,234)	4.7 (3,635,542)
Nervous system (N)	36.9 (17,985)	27.3 (37,443,242)	32.2 (6,835)	21.3 (12,600,000)	40.5 (11,150)	31.9 (24,700,000)
Analgesics (N02)	14.1 (6,848)	9.7 (13,364,618)	11.0 (2,328)	7.1 (4,212,587)	16.4 (4,520)	11.8 (9,107,326)
Antiepileptics (N03)	5.1 (2,461)	2.3 (3,124,887)	4.8 (1,018)	2.3 (1,354,867)	5.2 (1,443)	2.3 (1,758,064)
Psycholeptics (N05)	9.4 (4,563)	8.5 (11,614,648)	9.3 (1975)	6.9 (4,096,660)	9.4 (2,588)	9.7 (7,481,897)
Psychoanaleptics (N06)	7.1 (3,472)	5.6 (7,703,802)	6.1 (1,302)	3.9 (2,296,327)	7.9 (2,170)	7.0 (5,387,490)
Antiparasitic products, insecticides and repellents (P)	0.3 (134)	0.2 (301,954)	0.4 (84)	0.2 (101,466)	0.2 (50)	0.3 (198,061)
Respiratory system (R)	6.3 (3,084)	5.5 (7,577,337)	6.2 (1,323)	6.2 (3,661,700)	6.4 (1761)	5.0 (3,882,757)
Sensory organs (S)	1.6 (793)	6.2 (8,549,307)	1.2 (251)	5.7 (3,386,436)	2.0 (542)	6.6 (5,136,004)
Various (V)	0.2 (98)	0.1 (159,982)	0.3 (60)	0.2 (86,633)	0.1 (38)	0.1 (73,017)
HEALTHCARE PRODUCTS	2,276	Na	972	Na	1,304	Na
Wound dressings	47.4 (1,079)	Na	47.5 (462)	Na	47.3 (617)	Na
Surgical wound dressings	20.0 (456)	Na	27.9 (271)	Na	14.2 (185)	Na
Diapers	27.9 (636)	Na	20.4 (198)	Na	33.6 (438)	Na

^a^ The table only shows the therapeutic groups and type of healthcare products that represent at least a 5% of the total volume of medications and healthcare products, respectively. The whole table can be consulted in Supplementary Table 1.

Na = not available.

**TABLE 5:** Proportion of medicines and healthcare products used by age group, % (N).

	≤14		15–64		≥65	
	PB	Catalonia	PB	Catalonia	PB	Catalonia
Medicines, anatomical group (ATC level 1) and therapeutic group (ATC level 2)^a^	N = 2,417	N = 3,892,497	N = 40,378	N = 50,320,043	N = 8,228	N = 82,689,934
Alimentary tract and metabolism (A)	11.3 (235)	5.22 (203,126)	15.8 (6,161)	13.0 (6,533,202)	17.9 (1,392)	15.5 (12,800,000)
Drugs for acid related disorders (A02)	0.9 (18)	0.37 (14,432)	5.9 (2,279)	5.2 (2,596,489)	6.2 (480)	7.2 (5,911,230)
Drugs used in diabetes (A10)	0.2 (4)	0.53 (20,824)	6.2 (2,410)	4.7 (2,342,833)	8.6 (667)	5.3 (4,395,978)
Other alimentary tract and metabolism products (A16)	5.7 (120)	0.15 (5,962)	0 (0)	<0.1 (9,889)	0 (0)	<0.1 (2,762)
Blood and blood forming organs (B)	0.8 (16)	1.59 (61,871)	6.0 (2,315)	4.4 (2,201,112)	9.3 (721)	7.3 (6,023,645)
Antithrombotic agents (B01)	0 (0)	0.1 (3,948)	4.3 (1,664)	2.8 (1,397,537)	6.5 (507)	6.0 (4,945,210)
Cardiovascular system (C)	5.7 (120)	0.89 (34,789)	18.7 (7,286)	19.2 (9,654,331)	24.7 (1914)	29.8 (24,600,000)
Agents acting on the renin-angiotensin system (C09)	2.2 (46)	0.1 (3,756)	4.6 (1806)	7.0 (3,526,984)	7.2 (559)	9.5 (7,857,509)
Dermatologicals (D)	2.9 (60)	6.34 (246,778)	2.0 (768)	2.2 (1,117,447)	1.6 (123)	1.2 (991,620)
Genito urinary system and sex hormones (G)	0.4 (8)	0.22 (8,437)	1.2 (481)	2.8 (1,403,516)	2.3 (179)	2.8 (2,346,090)
Systemic hormonal preparations, excl. Sex hormones and insulins (H)	2.1 (43)	5.58 (217,191)	1.4 (558)	2.4 (1,180,885)	1.5 (119)	1.3 (1,102,044)
Antiinfectives for systemic use (J)	6.2 (129)	17.19 (668,951)	2.6 (1,012)	4.6 (2,332,305)	1.4 (106)	1.9 (1,552,050)
Antibacterials for systemic use (J01)	5.1 (107)	16.56 (644,641)	2.0 (759)	4.2 (2,087,746)	1.2 (93)	1.8 (1,448,848)
Antineoplastic and immunomodulating agents (L)	5.0 (105)	0.71 (27,603)	2.3 (878)	1.5 (743,741)	0.6 (46)	0.7 (589,730)
Immunosuppressants (L04)	5.0 (105)	0.48 (18,616)	2.2 (841)	1.1 (552,983)	0.3 (20)	0.4 (310,146)
Musculo-skeletal system (M)	3.3 (69)	11.91 (463,423)	4.2 (1,651)	6.9 (3,487,078)	2.0 (158)	2.8 (2,304,787)
Nervous system (N)	48.0 (1,004)	18.7 (727,716)	37.8 (14,713)	32.6 (16,400,000)	29.2 (2,268)	24.4 (20,200,000)
Analgesics (N02)	2.91 (61)	9.52 (370,691)	14.5 (5,644)	8.7 (4,400,211)	14.7 (1,143)	10.3 (8,549,011)
Antiepileptics (N03)	9.8 (206)	2.27 (88,513)	5.3 (2062)	3.7 (1,834,870)	2.5 (193)	1.4 (1,189,548)
Anti-Parkinson drugs (N04)	5.1 (107)	0.07 (2,687)	0.1 (51)	0.3 (171,035)	0.3 (21)	0.6 (489,661)
Psycholeptics (N05)	15.0 (314)	2.56 (99,491)	9.8 (3,804)	11.7 (5,888,111)	5.7 (445)	6.8 (5,590,955)
Psychoanaleptics (N06)	14.5 (303)	4.1 (159,702)	7.2 (2,794)	7.5 (3,761,175)	4.8 (375)	4.6 (3,762,940)
Antiparasitic products, insecticides and repellents (P)	0.2 (4)	1.16 (45,153)	0.3 (130)	0.4 (193,986)	0 (0)	0.1 (60,388)
Respiratory system (R)	12.7 (265)	15.46 (601,758)	6.3 (2,462)	6.5 (3,272,162)	4.6 (357)	4.4 (3,670,537)
Drugs for obstructive airway diseases (R03)	7.4 (154)	10.12 (393,914)	4.0 (1,544)	3.2 (1,622,557)	3.5 (268)	3.3 (2,714,370)
Sensory organs (S)	1.7 (35)	14.37 (559,340)	1.1 (412)	3.4 (1,715,146)	4.5 (346)	7.6 (6,247,954)
Various (V)	0 (0)	0.22 (8,459)	0.2 (67)	0.1 (58,570)	0.4 (31)	0.1 (92,621)
HEALTHCARE PRODUCTS	N = 324		N = 1,484		N = 468	
Wound dressings	12.0 (39)	Na	58.2 (863)	Na	37.82 (177)	Na
Surgical wound dressings	39.2 (127)	Na	18.9 (280)	Na	10.47 (49)	Na
Diapers	39.2 (127)	Na	18.7 (277)	Na	49.57 (232)	Na
Allergen-specific immunotherapy	7.1 (23)	Na	0.9 (13)	Na	0 (0)	Na

^a^ The table only shows the therapeutic groups and type of healthcare products that represent at least a 5% of the total volume of medications and healthcare products, respectively. The whole table can be consulted in Supplementary Table 2.

In comparison with the general Catalan population, among beneficiaries, medicines in the “cardiovascular system,” “genitourinary system and sex hormones,” and “sensory organs” anatomical groups represented a lower proportion of the total number of units of medications dispensed while medications in the “antineoplastic and immunomodulating agents” and “nervous system” groups represented a higher proportion (Table 4). Furthermore, in female beneficiaries, medicines for the “cardiovascular system” represented a lower proportion of medicines than in the general female population. The same was seen in males with medicines for the “genitourinary system and sex hormones” group (Table 4).

The largest variation in age groups was observed among children (0–14 years old) (Table 5). Compared with the general population of the same age group, medicines in the groups “alimentary tract and metabolism,” “cardiovascular system,” “antineoplastic and immunomodulating agents,” and “nervous system” represented a higher proportion (twice or higher) among beneficiaries than in the general population. In contrast, medicines in the “dermatological,” “systemic hormonal preparations,” “anti-infectives for systemic use,” “musculoskeletal system,” and “sensory organs” groups represented a lower proportion (half or less) among beneficiaries compared with the general population of children.

## Discussion

Beneficiaries were mainly adults, with functional disability and a low level of education, unemployed, and with one or more children. There is wide evidence regarding the relationships between the level of education, as a measure of socioeconomic position, and health (Furnee et al., 2008), and unemployment and health (Tøge and Blekesaune, 2015). The proportion of divorced people was higher in the general population, which could indicate lower social support for PB beneficiaries, compared with the rest of the population (Duffy, 1993; Kołodziej-Zaleska and Przybyła-Basista, 2016). The beneficiaries were polymedicated, and most were using medication for cardiovascular, mental, and pain-related disorders, which would also explain the high use of drugs for acid-related disorders, which are prescribed to prevent gastroduodenal side effects of polypharmacy and are generally overused (Forgacs and Loganayagam, 2008). Female beneficiaries were more likely to use medicines for pain-related and mental disorders than male beneficiaries, which could be related to a higher burden of chronic diseases in women (Malmusi et al., 2012).

The 12-month prevalence of use of psychotropic drugs (psycholeptics and psychoanaleptics) was high among PB beneficiaries (>42%) and higher than that reported in the Spanish primary care (Rubio-Valera et al., 2012). Socioeconomic status, debt, workplace conditions, and social capital are determinants of mental health (Bambra et al., 2010; Ahnquist et al., 2012; Sweet et al., 2013). This may partly explain the higher use of psychotropic drugs among PB beneficiaries, who were in a situation of economic hardship and more likely to be divorced than the general Catalan population. In addition, mental-health-related stigma increases vulnerability to unemployment, especially in males and individuals with low levels of education (Evans-Lacko et al., 2013). Thus, people with mental disorders may have experienced greater economic hardship than those without mental disorders following the economic crisis.

According to their profile of use of medicines and healthcare products and in contrast to a typical primary care pediatric patient, the pediatric population of beneficiaries presented severe chronic conditions. Thus, this population presents the most complex pediatric cases [such as heart diseases, autoimmune diseases, conduct disorders, and attention deficit hyperactivity disorder (ADHD)]. The complexity of these chronic and mental diseases may increase the vulnerability of the family, which has to cover medical costs (such as medication and private specialized care) and other costs (home adaptations, informal care, and educational accommodations) (McCann et al., 2012). In cases of economic hardship, children and adolescents are especially vulnerable (Rajmil et al., 2014). The high prevalence of use of these treatments among young beneficiaries could be explained by the greater prevalence of these disorders among disadvantaged populations (Reiss, 2013) but also by the high costs of these treatments. This vulnerability may be even more pronounced when the minor is presenting with severe diseases. It is necessary to evaluate the coverage of care needs for the child, especially when careers are experiencing financial problems. This would be the case, for instance, for approximately 30% of children and adolescents using psycholeptics and psychoanaleptics, usually indicated to treat conduct disorders and ADHD in the pediatric population.

More than half of the NGO beneficiaries were taking medications for pain-related disorders, and over a third were using opioids. Although there has been an increase in the use of opioids in Europe and the United States (Kalkman et al., 2019; Zhu et al., 2019), the prevalence of opioid use among PB beneficiaries is higher than that reported in previous studies. Similarly, the prevalence of use of other antiepileptics, which are also used to deal with pain-related disorders, was also high. Pain is highly disabling (Rice et al., 2016), and some opioids and antiepileptics, which are used in severe cases, are expensive. People with symptomatic and disabling disorders are more motivated to initiate a treatment (Gil-Girbau et al., 2019), which could partially explain why they seek help to cover the treatment expenses when they are experiencing financial hardship. However, chronic pain is associated with high productivity losses due to sick leave and unemployment, partly due to comorbidity with mood disorders (Dorner et al., 2016), which increases the vulnerability of people suffering from pain (Giladi et al., 2015). Low-wage workers and those with worse working conditions are at higher risk of developing pain-related disorders and experience barriers to accessing quality care, which could hinder recovery (Frederiksen et al., 2015; Wami et al., 2019).

Cardiovascular disease and mental disorders are both chronic disabling conditions with serious clinical and economic consequences (Vigo et al., 2016; Roth et al., 2017). Nonadherence to medication for cardiovascular disease and mental disorders worsens clinical status and increases the economic burden of these diseases (Lindström and Bingefors, 2000; Bitton et al., 2013; Ettehad et al., 2016). The cost of medications is of potential importance in adherence with medications for these conditions (Mathes et al., 2014), and the reduction of copayment increases adherence to chronic medications, especially among the more vulnerable populations (Choudhry et al., 2014; Simoens and Sinnaeve, 2014; Terraneo et al., 2014; Banerjee et al., 2016; Sinnott et al., 2016; Aznar-Lou et al., 2018). A high proportion of the beneficiaries were using medication for the primary and secondary prevention of cardiovascular diseases and diabetes as well as medication for mental disorders. However, the cost of these treatments is low and some patients in need for these treatments may not reach the 20 Euros or more per month threshold set to claim benefit from the SMF. Therefore, there may be a number of patients with no access to these treatments that have not yet been identified.

Economic burden increases the risk of developing several physical and mental disorders, which, in turn, could increase economic hardship, aggravating the situation of the patients experiencing chronic diseases and economic problems. Although the welfare system should ensure access to medicines for patients with economic problems, 30% of the beneficiaries renewed the aid, which could indicate chronification of pharmaceutical poverty. In the future, studies should be carried out to assess the prevalence of chronification of pharmaceutical poverty and its impact on health.

### Strengths and Limitations

Interpretation of the results of this study should take the following limitations into account. First, the sample comprises people who seek help and fulfill PB criteria to benefit from the SMF. Therefore, the results cannot be extrapolated to all patients in a situation of pharmaceutical poverty, such as those with acute conditions, homeless people and illegal immigrants. Second, the information came from a small geographically restricted area where the SMF was available in 2018. As the aid is extended to other areas, deeper insight will be gained. Third, the study was based on patient registries, and information on some key variables, such as comorbidities, social support, and medication dose, was missing.

In spite of these limitations, to the best of our knowledge, this is the first study to describe the pattern of use of medicines in a population affected by pharmaceutical poverty in a Western country. In this paper, we provide some clues about how morbidity patterns differ between the populations in situations of pharmaceutical poverty, which will be useful in better directing healthcare resources to vulnerable populations.

## Conclusion

Compared with the general population in Catalonia, patients presenting with pharmaceutical poverty were older and had a lower level of education, showed a higher prevalence of functional disability, were less likely to be Spanish, and were more likely to be divorced and unemployed. These patients commonly consumed treatments for pain-related disorders, gastrointestinal disorders, mental health–related disorders, and cardiovascular diseases.

Compared with male beneficiaries, females were more likely to use medication for pain-related disorders, gastrointestinal disorders, and mental health disorders and less likely to use medications for cardiovascular diseases and diabetes. Compared with the Catalan pediatric population, child and adolescent beneficiaries were more likely to use medicines for severe chronic diseases.

Decision makers should take these results into account when evaluating the existing copayment. A reformulation of the copayment law would be advisable. In the meantime, family physicians and pediatricians should explore economic barriers to access to treatment and direct their patients to resources to help cover the cost of their treatment, especially in the population at higher risk. Future studies should assess the impact of pharmaceutical poverty on health outcomes and explore this problem in populations presenting with acute conditions and in those that do not attend or do not have access to public healthcare centers, such as homeless people and illegal immigrants.

## Data Availability

The raw data supporting the conclusions of this article will be made available by the authors, without undue reservation, to any qualified researcher.

## Abbreviations

PB, Pharmaceutical Bank; WHO, World Health Organization; NHS, National Health System; NGOs, Non-Governmental Organizations; SMF, Social Medicine Fund; ESCA, Catalan Health Survey (*Enquesta de Salut de Catalunyua*, in Catalan); ATC, Anatomical Therapeutic Chemical; ADHD, Attention Deficit Hyperactivity Disorder.
