# Supplementary material for: Who Suffers From Pharmaceutical Poverty and What Are Their Needs? Evidence From a Spanish Region
Source: Front Pharmacol. 2021 Apr 20;12:617687. doi: 10.3389/fphar.2021.617687 (PMC8093809; doi:10.3389/fphar.2021.617687)
Supplement: Supplementary file 1 [file datasheet1.docx]

Supplementary Table 1. Proportion of beneficiaries that received at least one medicine or healthcare product overall and for males and females.

|  | Total (n=1206) |  | Females (n=623) |  | Males (n=583) |  |
| --- | --- | --- | --- | --- | --- | --- |
| **MEDICINES, Anatomical group (ATC level 1) and therapeutical group (ATC level 2)** | **N** | **%** | **N** | **%** | **N** | **%** |
| Alimentary tract and metabolism (A) | 817 | 67.7 | 460 | 73.84 | 357 | 61.23 |
| Stomatological preparations (A01) | 2 | 0.17 | 2 | 0.32 | 0 | 0 |
| Drugs for acid related disorders (A02) | 616 | 51.1 | 348 | 55.86 | 268 | 45.97 |
| Drugs for functional gastrointestinal disorders (A03) | 92 | 7.63 | 62 | 9.95 | 30 | 5.15 |
| Antiemetics and antinauseants (A04) | 21 | 1.74 | 15 | 2.41 | 6 | 1.03 |
| Bile and liver therapy (A05) | 12 | 1 | 8 | 1.28 | 4 | 0.69 |
| Drugs for constipation (A06) | 19 | 1.58 | 9 | 1.44 | 10 | 1.72 |
| Antidiarrheals, intestinal antiinflammatory/antiinfective agents (A07) | 40 | 3.32 | 29 | 4.65 | 11 | 1.89 |
| Digestives, incl. Enzymes (A09) | 4 | 0.33 | 2 | 0.32 | 2 | 0.34 |
| Drugs used in diabetes (A10) | 314 | 26 | 154 | 24.72 | 160 | 27.44 |
| Vitamins (A11) | 151 | 12.5 | 109 | 17.5 | 42 | 7.2 |
| Mineral supplements (A12) | 105 | 8.71 | 86 | 13.8 | 19 | 3.26 |
| Other alimentary tract and metabolism products (A16) | 1 | 0.08 | 1 | 0.16 | 0 | 0 |
| Blood and blood forming organs (B) | 495 | 41 | 238 | 38.2 | 257 | 44.08 |
| Antithrombotic agents (B01) | 371 | 30.8 | 152 | 24.4 | 219 | 37.56 |
| Antihemorrhagics (B02) | 4 | 0.33 | 4 | 0.64 | 0 | 0 |
| Antianemic preparations (B03) | 167 | 13.9 | 108 | 17.34 | 59 | 10.12 |
| Blood substitutes and perfusion solutions (B05) | 19 | 1.58 | 12 | 1.93 | 7 | 1.2 |
| Cardiovascular System (C) | 674 | 55.9 | 336 | 53.93 | 338 | 57.98 |
| Cardiac therapy (C01) | 135 | 11.2 | 37 | 5.94 | 98 | 16.81 |
| Antihypertensives (C02) | 34 | 2.82 | 11 | 1.77 | 23 | 3.95 |
| Diuretics (C03) | 180 | 14.9 | 96 | 15.41 | 84 | 14.41 |
| Peripheral vasodilators (C04) | 2 | 0.17 | 2 | 0.32 | 0 | 0 |
| Vasoprotectives (C05) | 8 | 0.66 | 3 | 0.48 | 5 | 0.86 |
| Beta blocking agents (C07) | 241 | 20 | 82 | 13.16 | 159 | 27.27 |
| Calcium channel blockers (C08) | 170 | 14.1 | 82 | 13.16 | 88 | 15.09 |
| Agents acting on the renin-angiotensin system (C09) | 434 | 36 | 218 | 34.99 | 216 | 37.05 |
| Lipid modifying agents (C10) | 466 | 38.6 | 205 | 32.91 | 261 | 44.77 |
| Dermatologicals (D) | 246 | 20.4 | 156 | 25.04 | 90 | 15.44 |
| Antifungals for dermatological use (D01) | 130 | 10.8 | 84 | 13.48 | 46 | 7.89 |
| Preparations for treatment of wounds and ulcers (D03) | 3 | 0.25 | 0 | 0 | 3 | 0.51 |
| Antipsoriatics (D05) | 15 | 1.24 | 10 | 1.61 | 5 | 0.86 |
| Antibiotics and chemotherapeutics for dermatological use (D06) | 36 | 2.99 | 20 | 3.21 | 16 | 2.74 |
| Corticosteroids, dermatological preparations (D07) | 119 | 9.87 | 76 | 12.2 | 43 | 7.38 |
| Anti-acne preparations (D10) | 7 | 0.58 | 5 | 0.8 | 2 | 0.34 |
| Other dermatological preparations (D11) | 17 | 1.41 | 11 | 1.77 | 6 | 1.03 |
| Genito urinary system and sex hormones (G) | 137 | 11.4 | 70 | 11.24 | 67 | 11.49 |
| Gynecological antiinfectives and antiseptics (G01) | 18 | 1.49 | 17 | 2.73 | 1 | 0.17 |
| Other gynecologicals (G02) | 4 | 0.33 | 3 | 0.48 | 1 | 0.17 |
| Sex hormones and modulators of the genital system (G03) | 25 | 2.07 | 25 | 4.01 | 0 | 0 |
| Urologicals (G04) | 96 | 7.96 | 30 | 4.82 | 66 | 11.32 |
| Systemic hormonal preparations, excl. Sex hormones and insulins (H) | 209 | 17.3 | 146 | 23.43 | 63 | 10.81 |
| Pituitary and hypothalamic hormones and analogues (H01) | 4 | 0.33 | 4 | 0.64 | 0 | 0 |
| Corticosteroids for systemic use (H02) | 124 | 10.3 | 82 | 13.16 | 42 | 7.2 |
| Thyroid therapy (H03) | 69 | 5.72 | 59 | 9.47 | 10 | 1.72 |
| Pancreatic hormones (H04) | 13 | 1.08 | 6 | 0.96 | 7 | 1.2 |
| Calcium homeostasis (H05) | 12 | 1 | 8 | 1.28 | 4 | 0.69 |
| Antiinfectives for systemic use (J) | 305 | 25.3 | 175 | 28.09 | 130 | 22.3 |
| Antibacterials for systemic use (J01) | 284 | 23.6 | 162 | 26 | 122 | 20.93 |
| Antimycotics for systemic use (J02) | 17 | 1.41 | 13 | 2.09 | 4 | 0.69 |
| Antimycobacterials (J04) | 7 | 0.58 | 3 | 0.48 | 4 | 0.69 |
| Antivirals for systemic use (J05) | 21 | 1.74 | 14 | 2.25 | 7 | 1.2 |
| Vaccines (J07) | 1 | 0.08 | 0 | 0 | 1 | 0.17 |
| Antineoplastic and immunomodulating agents (L) | 86 | 7.13 | 63 | 10.11 | 23 | 3.95 |
| Antineoplastic agents (L01) | 1 | 0.08 | 0 | 0.16 | 1 | 0 |
| Endocrine therapy (L02) | 17 | 1.41 | 16 | 2.57 | 1 | 0.17 |
| Immunosuppressants (L04) | 68 | 5.64 | 46 | 7.38 | 22 | 3.77 |
| Musculo-Skeletal System (M) | 821 | 68.1 | 242 | 38.84 | 143 | 24.53 |
| Antiinflammatory and antirheumatic products (M01) | 318 | 26.4 | 212 | 34.03 | 106 | 18.18 |
| Muscle relaxants (M03) | 35 | 2.9 | 22 | 3.53 | 13 | 2.23 |
| Antigout preparations (M04) | 40 | 3.32 | 11 | 1.77 | 29 | 4.97 |
| Drugs for treatment of bone diseases (M05) | 34 | 2.82 | 32 | 5.14 | 2 | 0.34 |
| Nervous System (N) | 952 | 78.9 | 525 | 84.27 | 427 | 73.24 |
| Anesthetics (N01) | 35 | 2.9 | 25 | 4.01 | 10 | 1.72 |
| Analgesics (N02) | 675 | 56 | 410 | 65.81 | 265 | 45.45 |
| Antiepileptics (N03) | 319 | 26.5 | 181 | 29.05 | 138 | 23.67 |
| Anti-parkinson drugs (N04) | 15 | 1.24 | 7 | 1.12 | 8 | 1.37 |
| Psycholeptics (N05) | 525 | 43.5 | 291 | 46.71 | 234 | 40.14 |
| Psychoanaleptics (N06) | 505 | 41.9 | 306 | 49.12 | 199 | 34.13 |
| Other nervous system drugs (N07) | 80 | 6.63 | 51 | 8.19 | 29 | 4.97 |
| Antiparasitic products, insecticides and repellents (P) | 19 | 1.58 | 11 | 1.77 | 8 | 1.37 |
| Respiratory System (R) | 387 | 32.1 | 212 | 34.03 | 175 | 30.02 |
| Nasal preparations (R01) | 115 | 9.54 | 64 | 10.27 | 51 | 8.75 |
| Drugs for obstructive airway diseases (R03) | 257 | 21.3 | 133 | 21.35 | 124 | 21.27 |
| Cough and cold preparations (R05) | 1 | 0.08 | 1 | 0.16 | 0 | 0 |
| Antihistamines for systemic use (R06) | 164 | 13.6 | 98 | 15.73 | 66 | 11.32 |
| Sensory Organs (S) | 161 | 13.4 | 99 | 15.89 | 62 | 10.63 |
| Ophthalmologicals (S01) | 144 | 11.9 | 87 | 13.96 | 57 | 9.78 |
| Otologicals (S02) | 21 | 1.74 | 15 | 2.41 | 6 | 1.03 |
| Ophthalmological and otological preparations (S03) | 2 | 0.17 | 1 | 0.16 | 1 | 0.17 |
| Various (V) | 22 | 1.82 | 13 | 2.09 | 9 | 1.54 |
| **HEALTHCARE PRODUCTS** | 226 | 18.74 | 129 | 20.71 | 97 | 16.64 |
| Wound dressings | 68 | 5.64 | 32 | 5.14 | 36 | 6.17 |
| Surgical wound dressings | 25 | 2.07 | 9 | 1.44 | 16 | 2.74 |
| Varicose vein stockings and socks | 7 | 0.58 | 5 | 0.8 | 2 | 0.34 |
| Inhalation chambers | 26 | 2.16 | 15 | 2.51 | 11 | 1.89 |
| Diapers | 94 | 7.79 | 63 | 10.11 | 31 | 5.32 |
| Allergen-specific immunotherapy | 35 | 2.9 | 14 | 2.25 | 21 | 3.6 |
| Other products | 13 | 1.08 | 8 | 1.28 | 5 | 0.86 |

Supplementary Table 2. Proportion of beneficiaries that received at least one medicine or healthcare product for age groups.

|  | ≤14 (n=104) |  | 15-64 (n=918) |  | ≥65 (n=184) |  |
| --- | --- | --- | --- | --- | --- | --- |
| **MEDICINES, Anatomical group (ATC level 1) and therapeutical group (ATC level 2)** | N | % | N | % | N | % |
| Alimentary tract and metabolism (A) | 14 | 13.33 | 647 | 70.56 | 156 | 84.78 |
| Stomatological preparations (A01) | 0 | 0 | 2 | 0.22 | 0 | 0 |
| Drugs for acid related disorders (A02) | 6 | 5.71 | 491 | 53.54 | 119 | 64.67 |
| Drugs for functional gastrointestinal disorders (A03) | 2 | 1.9 | 74 | 8.07 | 16 | 8.7 |
| Antiemetics and antinauseants (A04) | 1 | 0.95 | 19 | 2.07 | 1 | 0.54 |
| Bile and liver therapy (A05) | 0 | 0 | 10 | 1.09 | 2 | 1.09 |
| Drugs for constipation (A06) | 0 | 0 | 15 | 1.64 | 4 | 2.17 |
| Antidiarrheals, intestinal antiinflammatory/antiinfective agents (A07) | 3 | 2.86 | 33 | 3.6 | 4 | 2.17 |
| Digestives, incl. Enzymes (A09) | 0 | 0 | 4 | 0.44 | 0 | 0 |
| Drugs used in diabetes (A10) | 1 | 0.95 | 235 | 25.63 | 78 | 42.39 |
| Vitamins (A11) | 8 | 7.62 | 125 | 13.63 | 18 | 9.78 |
| Mineral supplements (A12) | 2 | 1.9 | 77 | 8.4 | 26 | 14.13 |
| Other alimentary tract and metabolism products (A16) | 1 | 0.95 | 0 | 0 | 0 | 0 |
| Blood and blood forming organs (B) | 8 | 7.62 | 379 | 41.33 | 108 | 58.7 |
| Antithrombotic agents (B01) | 0 | 0 | 277 | 30.21 | 94 | 51.09 |
| Antihemorrhagics (B02) | 0 | 0 | 3 | 0.33 | 1 | 0.54 |
| Antianemic preparations (B03) | 3 | 2.86 | 133 | 14.5 | 31 | 16.85 |
| Blood substitutes and perfusion solutions (B05) | 5 | 4.76 | 10 | 1.09 | 4 | 2.17 |
| Cardiovascular System (C) | 10 | 9.52 | 510 | 55.62 | 154 | 83.7 |
| Cardiac therapy (C01) | 3 | 2.86 | 105 | 11.45 | 27 | 14.67 |
| Antihypertensives (C02) | 4 | 3.81 | 19 | 2.07 | 11 | 5.98 |
| Diuretics (C03) | 0 | 0 | 133 | 14.5 | 47 | 25.54 |
| Peripheral vasodilators (C04) | 0 | 0 | 2 | 0.22 | 0 | 0 |
| Vasoprotectives (C05) | 0 | 0 | 8 | 0.87 | 0 | 0 |
| Beta blocking agents (C07) | 3 | 2.86 | 189 | 20.61 | 49 | 26.63 |
| Calcium channel blockers (C08) | 1 | 0.95 | 123 | 13.41 | 46 | 25 |
| Agents acting on the renin-angiotensin system (C09) | 2 | 1.9 | 317 | 34.57 | 115 | 62.5 |
| Lipid modifying agents (C10) | 0 | 0 | 354 | 38.6 | 112 | 60.87 |
| Dermatologicals (D) | 13 | 12.38 | 188 | 20.5 | 45 | 24.46 |
| Antifungals for dermatological use (D01) | 3 | 2.86 | 102 | 11.09 | 25 | 13.81 |
| Preparations for treatment of wounds and ulcers (D03) | 0 | 0 | 3 | 0.33 | 0 | 0 |
| Antipsoriatics (D05) | 0 | 0 | 13 | 1.41 | 2 | 1.1 |
| Antibiotics and chemotherapeutics for dermatological use (D06) | 4 | 3.81 | 28 | 3.04 | 4 | 2.21 |
| Corticosteroids, dermatological preparations (D07) | 8 | 7.62 | 90 | 9.78 | 21 | 11.6 |
| Anti-acne preparations (D10) | 1 | 0.95 | 5 | 0.54 | 1 | 0.55 |
| Other dermatological preparations (D11) | 4 | 3.81 | 12 | 1.3 | 1 | 0.55 |
| Genito urinary system and sex hormones (G) | 2 | 1.9 | 100 | 10.91 | 35 | 19.02 |
| Gynecological antiinfectives and antiseptics (G01) | 0 | 0 | 18 | 1.96 | 0 | 0 |
| Other gynecologicals (G02) | 0 | 0 | 4 | 0.43 | 0 | 0 |
| Sex hormones and modulators of the genital system (G03) | 0 | 0 | 25 | 2.72 | 0 | 0 |
| Urologicals (G04) | 2 | 1.9 | 60 | 6.52 | 34 | 18.78 |
| Systemic hormonal preparations, excl. Sex hormones and insulins (H) | 11 | 10.48 | 164 | 17.88 | 34 | 18.48 |
| Pituitary and hypothalamic hormones and analogues (H01) | 0 | 0 | 3 | 0.33 | 1 | 0.55 |
| Corticosteroids for systemic use (H02) | 8 | 7.62 | 99 | 10.76 | 17 | 9.39 |
| Thyroid therapy (H03) | 2 | 1.9 | 54 | 5.87 | 13 | 7.18 |
| Pancreatic hormones (H04) | 0 | 0 | 11 | 1.2 | 2 | 1.1 |
| Calcium homeostasis (H05) | 1 | 0.95 | 9 | 0.98 | 2 | 1.1 |
| Antiinfectives for systemic use (J) | 20 | 19.05 | 245 | 26.72 | 40 | 21.74 |
| Antibacterials for systemic use (J01) | 19 | 18.1 | 229 | 24.89 | 36 | 19.89 |
| Antimycotics for systemic use (J02) | 1 | 0.95 | 14 | 1.52 | 2 | 1.1 |
| Antimycobacterials (J04) | 0 | 0 | 7 | 0.76 | 0 | 0 |
| Antivirals for systemic use (J05) | 2 | 1.9 | 17 | 1.85 | 2 | 1.1 |
| Vaccines (J07) | 0 | 0 | 1 | 0.11 | 0 | 0 |
| Antineoplastic and immunomodulating agents (L) | 6 | 5.71 | 66 | 7.2 | 14 | 7.61 |
| Antineoplastic agents (L01) | 0 | 0 | 1 | 0.11 | 0 | 0 |
| Endocrine therapy (L02) | 0 | 0 | 12 | 1.3 | 5 | 2.76 |
| Immunosuppressants (L04) | 6 | 5.71 | 54 | 5.87 | 8 | 4.42 |
| Musculo-Skeletal System (M) | 23 | 21.9 | 317 | 34.57 | 45 | 24.46 |
| Antiinflammatory and antirheumatic products (M01) | 22 | 20.95 | 262 | 28.57 | 34 | 18.48 |
| Muscle relaxants (M03) | 1 | 0.95 | 34 | 3.71 | 0 | 0 |
| Antigout preparations (M04) | 1 | 0.95 | 31 | 3.38 | 8 | 4.35 |
| Drugs for treatment of bone diseases (M05) | 0 | 0 | 29 | 3.16 | 5 | 2.72 |
| Nervous System (N) | 64 | 60.95 | 736 | 80.26 | 152 | 82.61 |
| Anesthetics (N01) | 0 | 0 | 25 | 2.73 | 10 | 5.43 |
| Analgesics (N02) | 24 | 22.86 | 534 | 58.23 | 117 | 63.59 |
| Antiepileptics (N03) | 10 | 9.52 | 275 | 29.99 | 34 | 18.48 |
| Anti-parkinson drugs (N04) | 2 | 1.9 | 9 | 0.98 | 4 | 2.17 |
| Psycholeptics (N05) | 30 | 28.57 | 423 | 46.13 | 72 | 39.13 |
| Psychoanaleptics (N06) | 35 | 33.33 | 398 | 43.4 | 72 | 39.13 |
| Other nervous system drugs (N07) | 6 | 5.71 | 60 | 6.54 | 14 | 7.61 |
| Antiparasitic products, insecticides and repellents (P) | 3 | 2.86 | 16 | 1.74 | 0 | 0 |
| Respiratory System (R) | 38 | 36.19 | 289 | 31.52 | 60 | 32.61 |
| Nasal preparations (R01) | 15 | 14.29 | 86 | 9.38 | 14 | 7.61 |
| Drugs for obstructive airway diseases (R03) | 28 | 26.67 | 183 | 19.96 | 46 | 25 |
| Cough and cold preparations (R05) | 0 | 0 | 0 | 0 | 1 | 0.54 |
| Antihistamines for systemic use (R06) | 21 | 20 | 125 | 13.63 | 18 | 9.78 |
| Sensory Organs (S) | 13 | 12.38 | 103 | 11.23 | 45 | 24.46 |
| Ophthalmologicals (S01) | 12 | 11.43 | 89 | 9.67 | 43 | 23.76 |
| Otologicals (S02) | 1 | 0.95 | 16 | 1.74 | 4 | 2.21 |
| Ophthalmological and otological preparations (S03) | 0 | 0 | 2 | 0.22 | 0 | 0 |
| Various (V) | 0 | 0 | 20 | 2.18 | 2 | 1.09 |
| **HEALTHCARE PRODUCTS** | 40 | 38.1 | 132 | 14.39 | 54 | 29.35 |
| Wound dressings | 7 | 6.67 | 50 | 5.45 | 11 | 5.98 |
| Surgical wound dressings | 2 | 1.9 | 20 | 2.18 | 3 | 1.63 |
| Varicose vein stockings and socks | 0 | 0 | 6 | 0.65 | 1 | 0.54 |
| Inhalation chambers | 4 | 3.85 | 16 | 1.74 | 6 | 3.28 |
| Diapers | 11 | 10.48 | 44 | 4.8 | 39 | 21.2 |
| Allergen-specific immunotherapy | 22 | 20.95 | 13 | 1.42 | 0 | 0 |
| Other products | 3 | 2.86 | 10 | 1.09 | 0 | 0 |

Supplementary Table 3. Volume of medicines and healthcare products used by the beneficiaries and the general population in Catalonia overall and by gender.

|  | **Total** |  | **Males** |  | **Females** |  |
| --- | --- | --- | --- | --- | --- | --- |
|  | **PB** | **Catalonia** | **PB** | **Catalonia** | **PB** | **Catalonia** |
| **MEDICINES, Anatomical group (ATC level 1) and therapeutical group (ATC level 2)** | **48747** | **137378189** | **21225** | **59400000** | **27522** | **77500000** |
| Alimentary tract and metabolism (A) | 15.98 (7788) | 14.3 (19644551) | 15.9 (3375) | 14.52 (8632772) | 16.03 (4413) | 14.13 (10900000) |
| Stomatological preparations (A01) | <0.01 (2) | 0.02 (34313) | - | 0.02 (12877) | 0.01 (2) | 0.03 (20928) |
| Drugs for acid related disorders (A02) | 5.7 (2777) | 6.22 (8547897) | 5.35 (1136) | 6.01 (3571192) | 5.96 (1641) | 6.39 (4950959) |
| Drugs for functional gastrointestinal disorders (A03) | 0.88 (427) | 0.54 (746431) | 0.62 (132) | 0.41 (243593) | 1.07 (295) | 0.64 (498512) |
| Antiemetics and antinauseants (A04) | 0.19 (94) | 0.1 (136259) | 0.11 (23) | 0.07 (43935) | 0.26 (71) | 0.12 (91852) |
| Bile and liver therapy (A05) | 0.15 (73) | 0.08 (114057) | 0.08 (18) | 0.06 (33582) | 0.2 (55) | 0.1 (80172) |
| Drugs for constipation (A06) | 0.18 (88) | 0.08 (104943) | 0.23 (48) | 0.08 (48058) | 0.15 (40) | 0.07 (56793) |
| Antidiarrheals, intestinal antiinflammatory/antiinfective agents (A07) | 0.55 (269) | 0.51 (695264) | 0.72 (153) | 0.54 (323181) | 0.42 (116) | 0.48 (368953) |
| Digestives, incl. Enzymes (A09) | 0.05 (24) | 0.04 (56943) | 0.05 (11) | 0.06 (36562) | 0.05 (13) | 0.03 (20289) |
| Drugs used in diabetes (A10) | 6.32 (3081) | 4.93 (6777457) | 7.64 (1621) | 6.42 (3814420) | 5.3 (1460) | 3.8 (2945215) |
| Vitamins (A11) | 0.97 (473) | 0.74 (1012173) | 0.78 (166) | 0.47 (279012) | 1.12 (307) | 0.94 (725944) |
| Mineral supplements (A12) | 0.74 (360) | 1.02 (1400188) | 0.32 (67) | 0.37 (217153) | 1.06 (293) | 1.52 (1180284) |
| Other alimentary tract and metabolism products (A16) | 0.25 (120) | 0.01 (18622) | - | 0.02 (9204) | 0.44 (120) | 0.01 (9409) |
| Blood and blood forming organs (B) | 6.26 (3052) | 6.05 (8310079) | 8.06 (1711) | 7 (4163092) | 4.87 (1341) | 5.32 (4123536) |
| Antithrombotic agents (B01) | 4.45 (2171) | 4.63 (6364791) | 6.46 (1371) | 5.96 (3540321) | 2.91 (800) | 3.62 (2806374) |
| Antihemorrhagics (B02) | 0.02 (9) | 0.04 (56792) | - | 0.03 (15710) | 0.03 (9) | 0.05 (40851) |
| Antianemic preparations (B03) | 1.47 (719) | 1.27 (1750095) | 1.05 (222) | 0.91 (543360) | 1.81 (497) | 1.55 (1202002) |
| Blood substitutes and perfusion solutions (B05) | 0.31 (153) | 0.1 (138401) | 0.56 (118) | 0.11 (63701) | 0.13 (35) | 0.1 (74309) |
| Cardiovascular System (C) | 19.12 (9320) | 25.04 (34405442) | 24.88 (5281) | 28.98 (17200000) | 14.68 (4039) | 22.07 (17100000) |
| Cardiac therapy (C01) | 1.19 (578) | 0.93 (1272481) | 2.19 (464) | 1.2 (714188) | 0.41 (114) | 0.72 (555007) |
| Antihypertensives (C02) | 0.45 (219) | 0.47 (645757) | 0.73 (155) | 0.71 (424819) | 0.23 (64) | 0.28 (219617) |
| Diuretics (C03) | 2.38 (1162) | 3.55 (4875322) | 2.49 (529) | 3.21 (1907030) | 2.3 (633) | 3.82 (2958496) |
| Peripheral vasodilators (C04) | 0.01 (5) | 0.09 (128538) | - | 0.14 (85482) | 0.02 (5) | 0.05 (42503) |
| Vasoprotectives (C05) | 0.05 (26) | 0.04 (49249) | 0.04 (8) | 0.03 (16821) | 0.07 (18) | 0.04 (32172) |
| Beta blocking agents (C07) | 2.57 (1253) | 2.8 (3845270) | 3.88 (824) | 3.32 (1973379) | 1.56 (429) | 2.4 (1861766) |
| Calcium channel blockers (C08) | 1.7 (830) | 2.29 (3150722) | 1.95 (413) | 2.78 (1649564) | 1.52 (417) | 1.93 (1494407) |
| Agents acting on the renin-angiotensin system (C09) | 4.95 (2411) | 8.31 (11418280) | 5.9 (1252) | 9.57 (5685437) | 4.21 (1159) | 7.36 (5702812) |
| Lipid modifying agents (C10) | 5.82 (2836) | 6.57 (9019823) | 7.71 (1636) | 8.02 (4767132) | 4.36 (1200) | 5.46 (4229100) |
| Dermatologicals (D) | 1.95 (951) | 1.72 (2368761) | 1.43 (304) | 1.82 (1081384) | 2.35 (647) | 1.65 (1274461) |
| Antifungals for dermatological use (D01) | 0.96 (470) | 0.64 (873501) | 0.64 (136) | 0.63 (376950) | 1.21 (334) | 0.64 (492581) |
| Preparations for treatment of wounds and ulcers (D03) | 0.03 (16) | 0.01 (15223) | 0.08 (16) | 0.01 (5581) | - | 0.01 (9602) |
| Antipsoriatics (D05) | 0.09 (43) | 0.1 (142524) | 0.03 (6) | 0.15 (88010) | 0.13 (37) | 0.07 (54080) |
| Antibiotics and chemotherapeutics for dermatological use (D06) | 0.13 (64) | 0.25 (340584) | 0.11 (24) | 0.26 (156305) | 0.15 (40) | 0.23 (181563) |
| Corticosteroids, dermatological preparations (D07) | 0.58 (282) | 0.63 (863133) | 0.45 (95) | 0.65 (384803) | 0.68 (187) | 0.61 (473018) |
| Anti-acne preparations (D10) | 0.04 (18) | 0.05 (69450) | 0.01 (2) | 0.06 (34697) | 0.06 (16) | 0.04 (34461) |
| Other dermatological preparations (D11) | 0.12 (58) | 0.05 (64183) | 0.12 (25) | 0.06 (34942) | 0.12 (33) | 0.04 (29089) |
| Genito urinary system and sex hormones (G) | 1.37 (668) | 2.74 (3769794) | 1.74 (369) | 4.27 (2538678) | 1.09 (299) | 1.57 (1219365) |
| Gynecological antiinfectives and antiseptics (G01) | 0.07 (33) | 0.15 (201119) | <0.01 (1) | 0.01 (4948) | 0.12 (32) | 0.25 (194153) |
| Other gynecologicals (G02) | 0.02 (10) | 0.02 (20692) | 0.01 (3) | 0.01 (5790) | 0.03 (7) | 0.02 (14805) |
| Sex hormones and modulators of the genital system (G03) | 0.22 (106) | 0.56 (771985) | - | 0.12 (69570) | 0.39 (106) | 0.9 (699108) |
| Urologicals (G04) | 1.06 (519) | 2.02 (2775998) | 1.72 (365) | 4.14 (2458370) | 0.56 (154) | 0.4 (311299) |
| Systemic hormonal preparations, excl. Sex hormones and insulins (H) | 1.48 (720) | 1.83 (2510611) | 0.69 (146) | 1.28 (763523) | 2.09 (574) | 2.24 (1736597) |
| Pituitary and hypothalamic hormones and analogues (H01) | 0.04 (19) | 0.05 (61856) | - | 0.06 (34206) | 0.07 (19) | 0.04 (27485) |
| Corticosteroids for systemic use (H02) | 0.95 (461) | 0.88 (1205498) | 0.49 (105) | 0.91 (539287) | 1.29 (356) | 0.85 (659942) |
| Thyroid therapy (H03) | 0.31 (153) | 0.84 (1159626) | 0.1 (21) | 0.27 (158327) | 0.48 (132) | 1.29 (997420) |
| Pancreatic hormones (H04) | 0.05 (24) | 0.02 (30791) | 0.05 (11) | 0.03 (15221) | 0.05 (13) | 0.02 (15509) |
| Calcium homeostasis (H05) | 0.13 (63) | 0.04 (52840) | 0.04 (9) | 0.03 (16482) | 0.2 (54) | 0.05 (36241) |
| Antiinfectives for systemic use (J) | 2.56 (1247) | 3.35 (4598444) | 2.23 (473) | 3.27 (1942406) | 2.81 (774) | 3.37 (2610900) |
| Antibacterials for systemic use (J01) | 1.97 (959) | 3.07 (4223394) | 1.7 (361) | 3 (1781437) | 2.17 (598) | 3.1 (2399798) |
| Antimycotics for systemic use (J02) | 0.13 (65) | 0.11 (144802) | 0.06 (12) | 0.1 (57717) | 0.19 (53) | 0.11 (86117) |
| Antimycobacterials (J04) | 0.05 (22) | 0.02 (27998) | 0.07 (14) | 0.03 (15851) | 0.03 (8) | 0.02 (11933) |
| Antivirals for systemic use (J05) | 0.41 (199) | 0.1 (136617) | 0.4 (84) | 0.1 (58563) | 0.42 (115) | 0.1 (76780) |
| Vaccines (J07) | <0.01 (2) | 0.04 (57097) | 0.01 (2) | 0.05 (28660) | - | 0.04 (27974) |
| Antineoplastic and immunomodulating agents (L) | 2.11 (1029) | 0.99 (1364320) | 1.74 (369) | 0.94 (556179) | 2.4 (660) | 1.04 (804895) |
| Antineoplastic agents (L01) | <0.01 (1) | 0.08 (109697) | - | 0.09 (54617) | <0.01 (1) | 0.07 (54818) |
| Endocrine therapy (L02) | 0.13 (62) | 0.27 (369679) | <0.01 (1) | 0.12 (71917) | 0.22 (61) | 0.38 (296869) |
| Immunosuppressants (L04) | 1.98 (966) | 0.64 (883836) | 1.73 (368) | 0.72 (429012) | 2.17 (598) | 0.58 (452733) |
| Musculo-Skeletal System (M) | 3.85 (1878) | 4.58 (6293538) | 3.03 (644) | 4.41 (2619746) | 4.48 (1234) | 4.69 (3635542) |
| Antiinflammatory and antirheumatic products (M01) | 2.87 (1399) | 3.19 (4384430) | 1.9 (404) | 2.84 (1690125) | 3.62 (995) | 3.44 (2661495) |
| Muscle relaxants (M03) | 0.41 (201) | 0.28 (381820) | 0.6 (127) | 0.28 (166320) | 0.27 (74) | 0.28 (213382) |
| Antigout preparations (M04) | 0.27 (134) | 0.63 (864749) | 0.52 (110) | 1.16 (688211) | 0.09 (24) | 0.23 (174526) |
| Drugs for treatment of bone diseases (M05) | 0.3 (144) | 0.48 (662539) | 0.01 (3) | 0.13 (75090) | 0.51 (141) | 0.76 (586139) |
| Nervous System (N) | 36.89 (17985) | 27.26 (37443242) | 32.2 (6835) | 21.26 (12600000) | 40.51 (11150) | 31.86 (24700000) |
| Anesthetics (N01) | 0.26 (128) | 0.13 (176869) | 0.21 (44) | 0.09 (55631) | 0.31 (84) | 0.16 (120830) |
| Analgesics (N02) | 14.05 (6848) | 9.73 (13364618) | 10.97 (2328) | 7.09 (4212587) | 16.42 (4520) | 11.76 (9107326) |
| Antiepileptics (N03) | 5.05 (2461) | 2.27 (3124887) | 4.8 (1018) | 2.28 (1354867) | 5.24 (1443) | 2.27 (1758064) |
| Anti-parkinson drugs (N04) | 0.37 (179) | 0.48 (664743) | 0.22 (47) | 0.57 (340074) | 0.48 (132) | 0.42 (323309) |
| Psycholeptics (N05) | 9.36 (4563) | 8.45 (11614648) | 9.31 (1975) | 6.89 (4096660) | 9.4 (2588) | 9.66 (7481897) |
| Psychoanaleptics (N06) | 7.12 (3472) | 5.61 (7703802) | 6.13 (1302) | 3.86 (2296327) | 7.88 (2170) | 6.95 (5387490) |
| Other nervous system drugs (N07) | 0.69 (334) | 0.57 (781907) | 0.57 (121) | 0.47 (278033) | 0.77 (213) | 0.65 (500898) |
| Antiparasitic products, insecticides and repellents (P) | 0.27 (134) | 0.22 (301954) | 0.4 (84) | 0.17 (101466) | 0.18 (50) | 0.26 (198061) |
| Respiratory System (R) | 6.33 (3084) | 5.52 (7577337) | 6.23 (1323) | 6.16 (3661700) | 6.4 (1761) | 5.01 (3882757) |
| Nasal preparations (R01) | 0.62 (300) | 0.5 (687915) | 0.49 (105) | 0.57 (336687) | 0.71 (195) | 0.45 (347869) |
| Drugs for obstructive airway diseases (R03) | 4.03 (1966) | 3.46 (4747982) | 4.38 (929) | 4.21 (2502755) | 3.77 (1037) | 2.88 (2228086) |
| Cough and cold preparations (R05) | <0.01 (2) | 0.01 (9984) | - | 0.01 (5813) | 0.01 (2) | 0.01 (4128) |
| Antihistamines for systemic use (R06) | 1.67 (816) | 1.55 (2131456) | 1.36 (289) | 1.37 (816445) | 1.91 (527) | 1.68 (1302674) |
| Sensory Organs (S) | 1.63 (793) | 6.22 (8549307) | 1.18 (251) | 5.7 (3386436) | 1.97 (542) | 6.63 (5136004) |
| Ophthalmologicals (S01) | 1.55 (756) | 2.31 (3171289) | 1.15 (244) | 2.25 (1338858) | 1.86 (512) | 2.35 (1821238) |
| Otologicals (S02) | 0.07 (35) | 0.18 (251038) | 0.03 (6) | 0.19 (112342) | 0.11 (29) | 0.17 (133945) |
| Ophthalmological and otological preparations (S03) | <0.01 (2) | 0.01 (14865) | <0.01 (1) | 0.01 (6204) | <0.01 (1) | 0.01 (8523) |
| Various (V) | 0.2 (98) | 0.12 (159982) | 0.28 (60) | 0.15 (86633) | 0.14 (38) | 0.09 (73017) |
| **HEALTHCARE PRODUCTS** | 2276 |  | 972 |  | 1304 |  |
| Wound dressings | 47.41 (1079) | na | 47.53 (462) | na | 47.32 (617) | na |
| Surgical wound dressings | 20.04 (456) | na | 27.88 (271) | na | 14.19 (185) | na |
| Varicose vein stockings and socks | 0.83 (19) | na | 0.31 (3) | na | 1.23 (16) | na |
| Inhalation chambers | 1.14 (26) | na | 1.13 (11) | na | 1.15 (15) | na |
| Diapers | 27.94 (636) | na | 20.37 (198) | na | 33.59 (438) | na |
| Allergen-specific immunotherapy | 1.58 (36) | na | 2.16 (21) | na | 1.15 (15) | na |
| Other products | 1.05 (24) | na | 0.62 (6) | na | 1.38 (18) | na |

na = not available

Supplementary Table 4. Volume of medicines and healthcare products used by the beneficiaries and the general population in Catalonia by age group.

|  | ≤14 |  | 15-64 |  | ≥65 |  |
| --- | --- | --- | --- | --- | --- | --- |
|  | **PB** | **Catalonia** | **PB** | **Catalonia** | **PB** | **Catalonia** |
| **MEDICINES, Anatomical group (ATC level 1) and therapeutical group (ATC level 2)^$^** | **N=2417** | **N=3892497** | **N=40378** | **N=50320043** | **N=8228** | **N=82689934** |
| Alimentary tract and metabolism (A) | 11.23 (235) | 5.22 (203126) | 15.84 (6161) | 12.98 (6533202) | 17.94 (1392) | 15.53 (12800000) |
| Stomatological preparations (A01) | 0 (0) | 0.3 (11870) | 0.01 (2) | 0.02 (8654) | 0 (0) | 0.02 (13281) |
| Drugs for acid related disorders (A02) | 0.86 (18) | 0.37 (14432) | 5.86 (2279) | 5.16 (2596489) | 6.19 (480) | 7.15 (5911230) |
| Drugs for functional gastrointestinal disorders (A03) | 0.38 (8) | 0.3 (11545) | 0.82 (319) | 0.68 (339676) | 1.29 (100) | 0.47 (390884) |
| Antiemetics and antinauseants (A04) | 0.14 (3) | 0.05 (1879) | 0.21 (83) | 0.13 (62979) | 0.1 (8) | 0.09 (70929) |
| Bile and liver therapy (A05) | 0 (0) | 0.02 (945) | 0.18 (69) | 0.09 (47075) | 0.05 (4) | 0.08 (65734) |
| Drugs for constipation (A06) | 0 (0) | <0.01 (51) | 0.21 (82) | 0.08 (38792) | 0.08 (6) | 0.08 (66008) |
| Antidiarrheals, intestinal antiinflammatory/antiinfective agents (A07) | 0.24 (5) | 1.38 (53552) | 0.63 (245) | 0.64 (323511) | 0.24 (19) | 0.38 (315071) |
| Digestives, incl. Enzymes (A09) | 0 (0) | 0 (194) | 0.06 (24) | 0.05 (25728) | 0 (0) | 0.04 (30929) |
| Drugs used in diabetes (A10) | 0.19 (4) | 0.53 (20824) | 6.2 (2410) | 4.66 (2342833) | 8.6 (667) | 5.32 (4395978) |
| Vitamins (A11) | 3.39 (71) | 2.01 (78347) | 0.97 (376) | 0.76 (381186) | 0.34 (26) | 0.66 (545423) |
| Mineral supplements (A12) | 0.29 (6) | 0.09 (3525) | 0.7 (272) | 0.71 (356390) | 1.06 (82) | 1.25 (1037522) |
| Other alimentary tract and metabolism products (A16) | 5.73 (120) | 0.15 (5962) | 0 (0) | 0.02 (9889) | 0 (0) | <0.01 (2762) |
| Blood and blood forming organs (B) | 0.76 (16) | 1.59 (61871) | 5.95 (2315) | 4.37 (2201112) | 9.29 (721) | 7.28 (6023645) |
| Antithrombotic agents (B01) | 0 (0) | 0.1 (3948) | 4.28 (1664) | 2.78 (1397537) | 6.53 (507) | 5.98 (4945210) |
| Antihemorrhagics (B02) | 0 (0) | 0.06 (2315) | 0.02 (8) | 0.07 (36119) | 0.01 (1) | 0.02 (18127) |
| Antianemic preparations (B03) | 0.38 (8) | 0.73 (28486) | 1.57 (612) | 1.46 (735373) | 1.28 (99) | 1.19 (981503) |
| Blood substitutes and perfusion solutions (B05) | 0.38 (8) | 0.7 (27122) | 0.08 (31) | 0.06 (32083) | 1.47 (114) | 0.1 (78805) |
| Cardiovascular System (C) | 5.73 (120) | 0.89 (34789) | 18.73 (7286) | 19.19 (9654331) | 24.66 (1914) | 29.79 (24600000) |
| Cardiac therapy (C01) | 0.24 (5) | 0.28 (10932) | 1.17 (456) | 0.43 (214278) | 1.51 (117) | 1.26 (1043985) |
| Antihypertensives (C02) | 1.82 (38) | 0.31 (11878) | 0.35 (137) | 0.28 (141770) | 0.57 (44) | 0.59 (490788) |
| Diuretics (C03) | 0 (0) | 0.03 (1153) | 2.4 (933) | 1.94 (974874) | 2.95 (229) | 4.7 (3889499) |
| Peripheral vasodilators (C04) | 0 (0) | <0.01 (8) | 0.01 (5) | 0.05 (27471) | 0 (0) | 0.12 (100506) |
| Vasoprotectives (C05) | 0 (0) | <0.01 (17) | 0.07 (26) | 0.03 (17595) | 0 (0) | 0.04 (31381) |
| Beta blocking agents (C07) | 0.81 (17) | 0.09 (3602) | 2.62 (1019) | 2.18 (1096061) | 2.8 (217) | 3.31 (2735482) |
| Calcium channel blockers (C08) | 0.67 (14) | 0.03 (1191) | 1.62 (630) | 1.52 (766670) | 2.4 (186) | 2.87 (2376110) |
| Agents acting on the renin-angiotensin system (C09) | 2.2 (46) | 0.1 (3756) | 4.64 (1806) | 7.01 (3526984) | 7.2 (559) | 9.5 (7857509) |
| Lipid modifying agents (C10) | 0 (0) | 0.06 (2252) | 5.85 (2274) | 5.74 (2888628) | 7.24 (562) | 7.38 (6105352) |
| Dermatologicals (D) | 2.87 (60) | 6.34 (246778) | 1.97 (768) | 2.22 (1117447) | 1.59 (123) | 1.2 (991620) |
| Antifungals for dermatological use (D01) | 0.43 (9) | 1.36 (53090) | 1.02 (397) | 0.83 (418708) | 0.82 (64) | 0.48 (397733) |
| Preparations for treatment of wounds and ulcers (D03) | 0 (0) | <0.01 (56) | 0.04 (16) | 0.01 (2614) | 0 (0) | 0.02 (12513) |
| Antipsoriatics (D05) | 0 (0) | 0.02 (964) | 0.11 (41) | 0.18 (91441) | 0.03 (2) | 0.06 (49685) |
| Antibiotics and chemotherapeutics for dermatological use (D06) | 0.29 (6) | 2.04 (79365) | 0.13 (52) | 0.28 (142603) | 0.08 (6) | 0.14 (115900) |
| Corticosteroids, dermatological preparations (D07) | 1.43 (30) | 2.61 (101477) | 0.53 (206) | 0.76 (381853) | 0.59 (46) | 0.45 (374491) |
| Anti-acne preparations (D10) | 0.14 (3) | 0.19 (7582) | 0.04 (14) | 0.11 (55128) | 0.01 (1) | 0.01 (6448) |
| Other dermatological preparations (D11) | 0.57 (12) | 0.11 (4242) | 0.11 (42) | 0.05 (25024) | 0.05 (4) | 0.04 (34765) |
| Genito urinary system and sex hormones (G) | 0.38 (8) | 0.22 (8437) | 1.24 (481) | 2.79 (1403516) | 2.31 (179) | 2.84 (2346090) |
| Gynecological antiinfectives and antiseptics (G01) | 0 (0) | 0.07 (2577) | 0.08 (33) | 0.33 (168550) | 0 (0) | 0.03 (27974) |
| Other gynecologicals (G02) | 0 (0) | <0.01 (69) | 0.03 (10) | 0.04 (17821) | 0 (0) | <0.01 (2705) |
| Sex hormones and modulators of the genital system (G03) | 0 (0) | 0.07 (2545) | 0.27 (106) | 1.43 (721157) | 0 (0) | 0.05 (44976) |
| Urologicals (G04) | 0.38 (8) | 0.08 (3246) | 0.85 (332) | 0.99 (495988) | 2.31 (179) | 2.75 (2270435) |
| Systemic hormonal preparations, excl. Sex hormones and insulins (H) | 2.05 (43) | 5.58 (217191) | 1.43 (558) | 2.35 (1180885) | 1.53 (119) | 1.33 (1102044) |
| Pituitary and hypothalamic hormones and analogues (H01) | 0 (0) | 0.35 (13698) | 0.04 (15) | 0.06 (30815) | 0.05 (4) | 0.02 (17178) |
| Corticosteroids for systemic use (H02) | 1.43 (30) | 5.05 (196485) | 0.95 (371) | 0.92 (464224) | 0.77 (60) | 0.65 (538520) |
| Thyroid therapy (H03) | 0.24 (5) | 0.12 (4785) | 0.28 (110) | 1.32 (663324) | 0.49 (38) | 0.59 (487638) |
| Pancreatic hormones (H04) | 0 (0) | 0.06 (2191) | 0.05 (20) | 0.03 (13222) | 0.05 (4) | 0.02 (15317) |
| Calcium homeostasis (H05) | 0.38 (8) | <0.01 (32) | 0.11 (42) | 0.02 (9300) | 0.17 (13) | 0.05 (43391) |
| Antiinfectives for systemic use (J) | 6.16 (129) | 17.19 (668951) | 2.6 (1012) | 4.63 (2332305) | 1.37 (106) | 1.88 (1552050) |
| Antibacterials for systemic use (J01) | 5.11 (107) | 16.56 (644641) | 1.95 (759) | 4.15 (2087746) | 1.2 (93) | 1.75 (1448848) |
| Antimycotics for systemic use (J02) | 0.29 (6) | 0.06 (2204) | 0.15 (57) | 0.21 (103874) | 0.03 (2) | 0.05 (37756) |
| Antimycobacterials (J04) | 0 (0) | 0.04 (1435) | 0.06 (22) | 0.04 (18060) | 0 (0) | 0.01 (8289) |
| Antivirals for systemic use (J05) | 0.76 (16) | 0.08 (3213) | 0.44 (172) | 0.16 (78824) | 0.14 (11) | 0.06 (53306) |
| Vaccines (J07) | 0 (0) | 0.45 (17447) | 0.01 (2) | 0.07 (35538) | 0 (0) | <0.01 (3649) |
| Antineoplastic and immunomodulating agents (L) | 5.02 (105) | 0.71 (27603) | 2.26 (878) | 1.48 (743741) | 0.59 (46) | 0.71 (589730) |
| Antineoplastic agents (L01) | 0 (0) | 0.06 (2463) | <0.01 (1) | 0.06 (32636) | 0 (0) | 0.09 (74336) |
| Endocrine therapy (L02) | 0 (0) | 0.17 (6524) | 0.09 (36) | 0.31 (157234) | 0.34 (26) | 0.25 (205028) |
| Immunosuppressants (L04) | 5.02 (105) | 0.48 (18616) | 2.16 (841) | 1.1 (552983) | 0.26 (20) | 0.38 (310146) |
| Musculo-Skeletal System (M) | 3.3 (69) | 11.91 (463423) | 4.24 (1651) | 6.93 (3487078) | 2.04 (158) | 2.79 (2304787) |
| Antiinflammatory and antirheumatic products (M01) | 2.68 (56) | 11.76 (457765) | 3.16 (1228) | 5.58 (2810171) | 1.48 (115) | 1.31 (1083684) |
| Muscle relaxants (M03) | 0.57 (12) | 0.13 (4934) | 0.49 (189) | 0.56 (282541) | 0 (0) | 0.11 (92227) |
| Antigout preparations (M04) | 0.05 (1) | 0.02 (700) | 0.27 (106) | 0.48 (241597) | 0.35 (27) | 0.75 (620440) |
| Drugs for treatment of bone diseases (M05) | 0 (0) | <0.01 (24) | 0.33 (128) | 0.3 (152769) | 0.21 (16) | 0.61 (508436) |
| Nervous System (N) | 47.97 (1004) | 18.7 (727716) | 37.83 (14713) | 32.57 (16400000) | 29.23 (2268) | 24.43 (20200000) |
| Anesthetics (N01) | 0 (0) | 0.14 (5536) | 0.29 (112) | 0.1 (49489) | 0.21 (16) | 0.15 (121436) |
| Analgesics (N02) | 2.91 (61) | 9.52 (370691) | 14.51 (5644) | 8.74 (4400211) | 14.73 (1143) | 10.34 (8549011) |
| Antiepileptics (N03) | 9.84 (206) | 2.27 (88513) | 5.3 (2062) | 3.65 (1834870) | 2.49 (193) | 1.44 (1189548) |
| Anti-parkinson drugs (N04) | 5.11 (107) | 0.07 (2687) | 0.13 (51) | 0.34 (171035) | 0.27 (21) | 0.59 (489661) |
| Psycholeptics (N05) | 15 (314) | 2.56 (99491) | 9.78 (3804) | 11.7 (5888111) | 5.73 (445) | 6.76 (5590955) |
| Psychoanaleptics (N06) | 14.48 (303) | 4.1 (159702) | 7.18 (2794) | 7.47 (3761175) | 4.83 (375) | 4.55 (3762940) |
| Other nervous system drugs (N07) | 0.62 (13) | 0.03 (1096) | 0.63 (246) | 0.56 (283305) | 0.97 (75) | 0.6 (494530) |
| Antiparasitic products, insecticides and repellents (P) | 0.19 (4) | 1.16 (45153) | 0.33 (130) | 0.39 (193986) | 0 (0) | 0.07 (60388) |
| Respiratory System (R) | 12.66 (265) | 15.46 (601758) | 6.33 (2462) | 6.5 (3272162) | 4.6 (357) | 4.44 (3670537) |
| Nasal preparations (R01) | 1.29 (27) | 1.27 (49472) | 0.62 (242) | 0.8 (401257) | 0.4 (31) | 0.28 (233827) |
| Drugs for obstructive airway diseases (R03) | 7.36 (154) | 10.12 (393914) | 3.97 (1544) | 3.22 (1622557) | 3.45 (268) | 3.28 (2714370) |
| Cough and cold preparations (R05) | 0 (0) | 4.07 (158372) | 0 (0) | 0.01 (3426) | 0.03 (2) | 0.01 (6515) |
| Antihistamines for systemic use (R06) | 4.01 (84) |  | 1.74 (676) | 2.47 (1244922) | 0.72 (56) | 0.87 (715825) |
| Sensory Organs (S) | 1.67 (35) | 14.37 (559340) | 1.06 (412) | 3.41 (1715146) | 4.46 (346) | 7.56 (6247954) |
| Ophthalmologicals (S01) | 1.62 (34) | 3.68 (143347) | 0.99 (385) | 1.55 (781217) | 4.34 (337) | 2.7 (2235532) |
| Otologicals (S02) | 0.05 (1) | 2.03 (79133) | 0.06 (25) | 0.23 (117630) | 0.12 (9) | 0.06 (49524) |
| Ophthalmological and otological preparations (S03) | 0 (0) | 0.04 (1419) | 0.01 (2) | 0.02 (7767) | 0 (0) | 0.01 (5541) |
| Various (V) | 0 (0) | 0.22 (8459) | 0.17 (67) | 0.12 (58570) | 0.4 (31) | 0.11 (92621) |
| **HEALTHCARE PRODUCTS** | **N=324** |  | **N=1484** |  | **N=468** |  |
| Wound dressings | 12.04 (39) | na | 58.15 (863) | na | 37.82 (177) | na |
| Surgical wound dressings | 39.2 (127) | na | 18.87 (280) | na | 10.47 (49) | na |
| Varicose vein stockings and socks | 0 (0) | na | 1.01 (15) | na | 0.85 (4) | na |
| Inhalation chambers | 1.23 (4) | na | 1.08 (16) | na | 1.28 (6) | na |
| Diapers | 39.2 (127) | na | 18.67 (277) | na | 49.57 (232) | na |
| Allergen-specific immunotherapy | 7.1 (23) | na | 0.88 (13) | na | 0 (0) | na |
| Other products | 1.23 (4) | na | 1.35 (20) | na | 0 (0) | na |

na: not available
